# Integration of 1:1 orthology maps and updated datasets into Echinobase

**DOI:** 10.1093/database/baab030

**Published:** 2021-06-26

**Authors:** Saoirse Foley, Carolyn Ku, Brad Arshinoff, Vaneet Lotay, Kamran Karimi, Peter D Vize, Veronica Hinman

**Affiliations:** Department of Biological Sciences, Carnegie Mellon University, 5000 Forbes Avenue, Pittsburgh, PA 15213, USA; Echinobase #6-46, Mellon Institute, 4400 Fifth Avenue, Pittsburgh, PA 15213, USA; Department of Biological Sciences, Carnegie Mellon University, 5000 Forbes Avenue, Pittsburgh, PA 15213, USA; Echinobase #6-46, Mellon Institute, 4400 Fifth Avenue, Pittsburgh, PA 15213, USA; Department of Biological Sciences, University of Calgary, 2500 University Drive NW, Calgary, Alberta TN2 1N4, Canada; Department of Biological Sciences, University of Calgary, 2500 University Drive NW, Calgary, Alberta TN2 1N4, Canada; Department of Biological Sciences, University of Calgary, 2500 University Drive NW, Calgary, Alberta TN2 1N4, Canada; Department of Biological Sciences, University of Calgary, 2500 University Drive NW, Calgary, Alberta TN2 1N4, Canada; Department of Biological Sciences, Carnegie Mellon University, 5000 Forbes Avenue, Pittsburgh, PA 15213, USA; Echinobase #6-46, Mellon Institute, 4400 Fifth Avenue, Pittsburgh, PA 15213, USA

## Abstract

Echinobase (https://echinobase.org) is a central online platform that generates, manages and hosts genomic data relevant to echinoderm research. While the resource primarily serves the echinoderm research community, the recent release of an excellent quality genome for the frequently studied purple sea urchin (*Strongylocentrotus purpuratus* genome, v5.0) has provided an opportunity to adapt to the needs of a broader research community across other model systems. To this end, establishing pipelines to identify orthologous genes between echinoderms and other species has become a priority in many contexts including nomenclature, linking to data in other model organisms, and in internal functionality where data gathered in one hosted species can be associated with genes in other hosted echinoderms. This paper describes the orthology pipelines currently employed by Echinobase and how orthology data are processed to yield 1:1 ortholog mappings between a variety of echinoderms and other model taxa. We also describe functions of interest that have recently been included on the resource, including an updated developmental time course for *S.purpuratus*, and additional tracks for genome browsing. These data enhancements will increase the accessibility of the resource to non-echinoderm researchers and simultaneously expand the data quality and quantity available to core Echinobase users.

**Database URL**: https://echinobase.org

## Introduction

Members of Echinodermata, which includes sea urchins, sea stars, sea cucumbers and crinoids, represent excellent model systems to address emergent research questions across biology, including gene regulatory network evolution (1), developmental patterning (2), skeletogenesis (3) and regeneration (4). Echinobase (5) generates, organizes and hosts a wealth of data associated with echinoderm genomics. The resource allows users to access raw sequence data, gene and protein information, gene expression data as well as laboratory materials (e.g. Bacterial Artificial Chromosome (BAC) libraries, probe hybridization protocols and experimental workflows).

One limitation of the resource in recent years has been the absence of high-quality echinoderm genomes. This has impeded the production of accurate orthology maps between echinoderms and other species. Orthologs refer to genes in different species that are derived via speciation, as opposed to paralogs that arise via duplication events (6). Given their shared ancestral history, orthologous genes are commonly used in the construction of phylogenies and can be putatively considered to share similar functions, with some caveats (7–9). These relationships also serve less visible roles in the web applications that drive the Echinobase database functionality. When genes in different echinoderm species are mapped as orthologs, data curated to a gene in one species are linked to the orthologous gene via a database relationship. In this manner, data such as a gene expression pattern or Gene Ontology (GO) annotations associated with a *Strongylocentrotus purpuratus* (purple sea urchin) gene are also linked to the orthologous gene in other Echinobase species, such as the sea stars *Acathaster planci* and *Patiria miniata*. Sparse data against one species, for example, papers on the developmental biology of *Acanthaster*, can thereby be directly linked to the rich data available on this subject in *S. purpuratus*.

**Figure 1.**
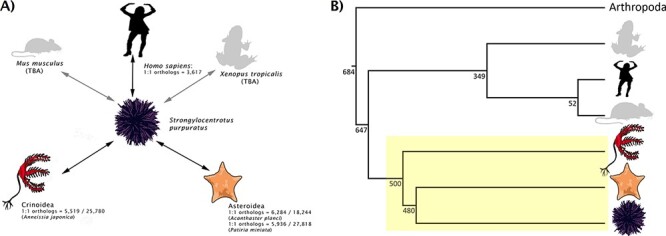
(A) Rationale behind taxon choice for orthology analyses at Echinobase. Black arrows represent analyses that have been performed. We have so far used *Anneissia japonica* for Crinoidea analysis and both *Acanthaster planci* and *Patiria miniata* for Asteroidea. Gray arrows show analyses planned for integration (e.g. between *S. purpuratus and Mus musculus, Xenopus tropicalis*). This demonstrates our use as *S. purpuratus* as our reference echinoderm, in that orthologs can be laterally inferred between other echinoderms and non-echinoderms via *S. purpuratus*. 1:1 ortholog counts for intra-echinoderm analyses are expressed as fractions of the total number of genes in the genome of that species. (B) A species tree, showing the phylogenetic positions of different model metazoan taxa relative to echinoderms (box highlighted in yellow). Branch lengths are not drawn to scale. Numbers beside the nodes are estimated times in millions of years which, along with the topology, are adapted from the studies by Cary and Hinman, Delsuc *et al*. and Dohrmann and Wörheide (34–36). Echinoderms are an extremely diverse group; different classes of echinoderm are distantly related to each other.

The recent *S. purpuratus* v5.0 genome release is hosted on Echinobase and is of excellent quality, with NG50 scores of ∼37 Mb. This has allowed Echinobase to establish orthology pipelines, with the end goal being to emulate a system similar to that on the DRSC Integrative Ortholog Prediction Tool (i.e. a DIOPT-like system), consisting of several different tools to identify orthologs both within echinoderm species and between echinoderms and human genes for the purpose of gene nomenclature and gene function modeling. While clear 1:1 orthology relationships typically do not represent >20–35% of the total number of genes in the genome (e.g. Figure 1), a full ortholog map between *S. purpuratus* and other major model organisms is not yet possible due to the absence of the v5.0 urchin genome in numerous external resources that are essential for a full DIOPT-like analysis, e.g. UniProt and PANTHER. While we are making progress toward *S. purpuratus* integration with these resources and more, the computational resources for such a mapping are extensive and will have to await broader incorporation of echinoderm data into these resources. As a temporary method to link out to orthologs in other model organisms, we use non-curated National Center for Biotechnology Information (NCBI) ortholog data to provide best guess links to other model species and use the higher-quality (but less broad) in-house mapping predictions described herein to drive database functionality.

For the purpose of generating orthology maps to build relationships between data for different represented echinoderm species, and given the assembly quality, the Echinobase team decided that *S. purpuratus* v5.0 should constitute our model echinoderm and serve as our anchor between echinoderms and human genomic data. Hence, the focus of our orthology analyses will be to identify orthologs between *S. purpuratus* and other organisms (e.g. *Homo sapiens*) and also between *S. purpuratus* and other echinoderms in Echinobase (e.g. *Acanthaster planci*). In this way, orthologs between other echinoderms and non-echinoderms can be laterally inferred through *S. purpuratus* (Figure 1). Although this approach will result in the population of more data fields for *S. purpuratus* relative to the other echinoderms on Echinobase, it facilitates the attachment of data to those echinoderms even if a 1:1 human ortholog would not be recovered. The primary reason for selecting *S. purpuratus* as the anchor species, however, is due to the quality of the genome and the major effort at annotating its gene models, including a community annotation jamboree by domain experts (11). This species also represents a large portion of experimental papers and data out from high-throughput screens and hence has a rich set of biological data supporting the annotation. Similar supporting content is not yet available for other echinoderm species supported by Echinobase. This approach is similar to that used by other Model Organism Databases (i.e. MODs), such as Flybase that uses the major experimental model *Drosophila melanogaster* as its core species. As there are no strong reasons not to use this *S. purpuratus* as the anchor in Echinobase while the above-mentioned reasons support its adoption, we proceeded accordingly. This logic means that two echinoderms that have more similar genomes to each other than they do to the human genome can share associated data in Echinobase, while such content would often be lost if relationships were built between each echinoderm and human (e.g. if relating two echinoderms required transiting via human relationship).

In this paper, we report on our progress toward establishing a DIOPT-like system to identify unique 1:1 orthologs and implement codes that use these relationships to link data in the different represented species. These 1:1 mappings are used by Echinobase to drive user features and the assembly of gene pages, such as the multi-species view under the ‘Molecule’ section of Gene Pages. A separate ‘Ortholog’ section in Echinobase provides NCBI predicted orthologs in other model organisms, but these data are not incorporated in the database and these non-curated relationships only link to external resources. We discuss the orthology tools integrated into our pipeline to date and review the progress made toward integrating *S. purpuratus* v5.0 with other external databases and orthology tools. Lastly, we describe updates to our quantification of developmental transcript profiles in *S. purpuratus* from RNA-Seq data, as well as the inclusion of more tracks into the on-site genome browser.

## Identifying 1:1 orthologous genes

The Alliance of Genome Resources (12) states that pairs of genes may be considered orthologous if three or more of the 12+ tools in the DIOPT suite converge upon the result. Thus far, Echinobase pipelines have been established to run the following tools under stringent conditions: InParanoid v4.1 (13, 14), ProteinOrtho v6 (15), SwiftOrtho (16) and FastOrtho (http://enews.patricbrc.org/) were run with e-values set to 1e-40 in each case, with FastOrtho’s inflation parameter set to 1.5; OMA v2.4.1 (17) and OrthoFinder v2.4 (18) were run under default conditions.

These tools were implemented to identify orthologs between *S. purpuratus* and each of *Anniessia japonica* (feather star, v1.0), *P.miniata* (3.0) and *A.planci* (crown-of-thorns sea star, v1.0) for a lateral pan-phyletic sampling of Echinodermata, as well as *Homo sapiens* (human, UP000005640) to inform downstream nomenclature pipelines (Beatman *et al.*, in preparation) and for integration into model organism resources.

The outputs from each tool are represented by groups of orthologous hits, where orthogroups consisting of proteins are each listed on their own line. Once a full suite of outputs is received for a given pair of species, each tool’s output is subjected to several rounds of filtering and processing. First, given that many of these tools can also predict paralogs, orthogroups that contain entries from only single-species hits are removed. Each protein in each orthogroup hit is then stripped to ensure that only the RefSeq ID remains. Next, given that many RefSeq IDs can correspond to a single Entrez gene ID, the RefSeq IDs are converted to Entrez IDs using a combination of keys found in the GTF files for each taxon and GPFF files hosted at the NCBI RefSeq database (https://ftp.ncbi.nlm.nih.gov/refseq/release/invertebrate/). A series of in-house Python scripts are then used to group and collapse any duplicate Entrez entries now present in the output, yielding a series of orthogroups where each Entrez ID is only represented once per output. At this point, co-orthologs can be identified by cases where multiple Entrez entries for a given species are present in an orthogroup. 1:1 ortholog predictions are identified as orthogroups that contain only a single Entrez entry per species, as the collapsing and grouping process has ensured that these entries do not have additional orthologs beyond the reported orthogroup. This process is repeated for each tool output, and a 1:1 orthology call can be made by Echinobase in cases where three or more tools each contain the same orthogroup with only a single Entrez entry per species. This pipeline may be repeated for any given pair of taxa. However, our web application uses relationships to the core species (*S. purpuratus*) to associate data, so current orthology predictions between other species presently do not play a role in code logic.

## New JBrowse tracks and an updated S. purpuratus v5.0 developmental time course

Tu *et al.* generated RNA-Seq data across 10 different developmental timepoints in *S.purpuratus*, ranging from 0 to 72 hours post-fertilization. These data were downloaded from the SRA archive (bioproject number: PRJNA81157). Sequencing adapters were trimmed from the reads using TrimGalore v0.6.3 (21). The updated *S.purpuratus* v5.0 genome file along with its associated GTF were downloaded from Echinobase (5).

RSEM v1.2.21 (22) was used in tandem with Bowtie 2 v2.3.4.1 (23) to prepare an initial RSEM reference input. Expression was quantified in RSEM using two different metrics: transcripts per million (TPM) and fragments per kilobase million (FPKM). As per Tu *et al.* (20), the FPKM values were multiplied by 60 to yield transcripts per embryo (TPE) values. This method also yields a BAM file in genomic coordinates for each timepoint in addition to the expression values. These BAMs were sorted and indexed using SAMtools v1.9 (24) and subsequently converted to the more compact bigWig format using deepTools v2.3.5 (25). The TPE data are stored as read count data directly in the database and are processed and displayed dynamically via the tools previously built in Xenbase [as detailed in the study by Fortriede *et al.* (26)].

Whole genome Assay for Transposase-Accessible Chromatin (i.e. ATAC)-Seq data were also downloaded from the SRA archive (bioproject number: PRJNA377768) corresponding to three replicate *S. purpuratus* embryo cultures. Each replicate culture was sampled at seven different timepoints, ranging from 18 to 70 hours post-fertilization, representing a time course of open chromatin through embryogenesis. These 21 ATAC-Seq samples were aligned to the *S. purpuratus* v5.0 genome file.

TrimGalore v0.6.3 (21) was used to trim adapter sequences from the reads, which were then aligned to the *S. purpuratus* v5.0 genome with Bowtie2 v2.3.4.1 (23). Alignments were filtered and sorted using SAMtools v1.9 (24), and the alignment files were converted to BED format with BEDtools v2.25.0 (27). These BED tools were used for peak calling with MACS2 v2.1.1 (28). The resulting peak files of the three replicates for each time point were merged. These peak files were also converted to bigWig using deepTools v2.5.3 (25), which displays the score of each peak as a peak height in the genome browser.

## Data accession

**Figure 2.**
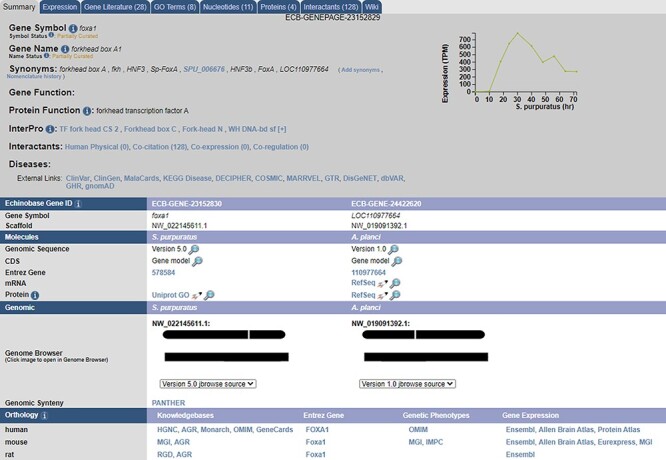
A sample gene summary page for foxa1. The updated developmental time course is shown in the top right. The ortholog of the gene in different echinoderms, as predicted by our pipeline, is seen under the ‘Echinobase Gene ID’ section. The gene can be viewed in JBrowse by clicking the links under the ‘Genomic’ section. Additional orthologs to non-echinoderm species as predicted by NCBI and are reported under the ‘Orthology’ section.

1:1 ortholog data between *S. purpuratus* and each of *A. planci, P. miniata, A. japonica* and *H. sapiens* are summarized in Figure 1 and hosted on Echinobase (5). Six thousand two hundred and eighty-four 1:1 orthologs were recovered in *A. planci*, 5936 in *P. miniata*, 5519 in *A. japonica* and 3617 in *H. sapiens*. The raw outputs from each tool along with the consensus 1:1 mappings for each analysis can be accessed from the Echinobase FTP site (http://ftp.echinobase.org/pub/Orthology/). Both ortholog data and a gene expression time course can be found on individual gene pages (Figure 2). While Echinobase previously reported TPE expression data from external sources, we took this opportunity to start reporting expression in TPM values, as it is now more commonly used and considered to be less biased than metrics derived from FPKM values (29). Raw TPM and TPE values can also be downloaded from the Echinobase FTP site (http://ftp.echinobase.org/pub/Expression/S.pur/).

**Figure 3.**
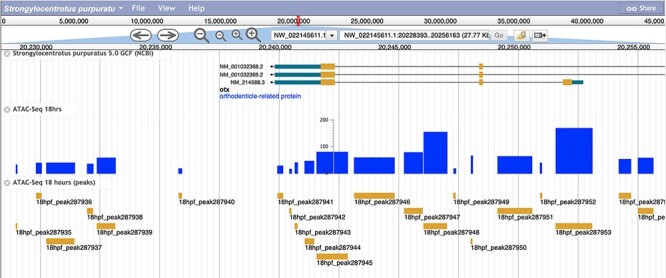
A screenshot of the *S. purpuratus* v5.0 genome browser displaying the labeled 18 hours post-fertilization ATAC-seq peaks and peak scores.

The associated bigWig tracks for both the RNA-Seq and ATAC-Seq peaks at each associated timepoint have been integrated into the JBrowse Genome viewer and can be accessed from the Echinobase homepage by clicking on the ‘Genomes’ tab and subsequently selecting ‘*S. purpuratus* 5.0’ (Figure 3).

## Future perspectives

We have taken several steps to integrate the *S.purpuratus* v5.0 data with other resources. When realized, these steps will gradually increase the ortholog counts reported in Figure 1. First, as of the latest update, Ensembl Metazoa v49 has integrated of *S. purpuratus* v5.0, which is now represented in its portal. This will allow additional database-driven orthology tools to access the updated version *S. purpuratus* and have it represented in their outputs. We have confirmed this with the developers of several tools, including PANTHER (30) and TreeFAM (31). OrthoMCL (32) have also agreed to include *S. purpuratus* as a peripheral species, with a view toward eventually including it as a core species once the data are integrated into UniProt. Lastly, we are currently working with PhylomeDB developers (33) to generate reference phylomes for *S. purpuratus*. Non-echinoderm researchers can also benefit from these efforts, as these data will be more accessible to the broader scientific community.

Given that our analyses are run under stringent homology prediction conditions (e.g. 1e-40 during initial BLAST phases), the 1:1 ortholog data recovered here should remain stable as more tools are added to our pipeline. This has allowed the nomenclature team at Echinobase to begin annotating these genes via their nomenclature pipelines (Beatman *et al.*, (19)) according to the echinoderm gene nomenclature guidelines (https://www.echinobase.org/gene/static/geneNomenclature.jsp).

We also plan to resolve many:1 orthologies once enough tools have been run such that we can emulate a DIOPT-like (10) system—i.e. at least 12 tools. This decision was made to preserve the integrity of our orthology predictions, as the agreed-upon consensus of the true ortholog in many:1 cases may shift as more tools are added. We will be following the guidelines set by the Alliance of Genome Resources (12) at that time.
